# Reevaluation of NOD/SCID Mice as NK Cell-Deficient Models

**DOI:** 10.1155/2021/8851986

**Published:** 2021-11-10

**Authors:** Miao Miao, Henry Masengere, Guang Yu, Fengping Shan

**Affiliations:** ^1^Department of Immunology, School of Basic Medical Science, China Medical University, Shenyang 110122, China; ^2^Department of Immunology, School of Basic Medical Science, Jinzhou Medical University, Jinzhou 121001, China

## Abstract

**Objective:**

Natural killer (NK) cell-deficient mice are useful models in biomedical research. NOD/SCID mice have been used as a model of this type in research. However, the actual status of NK cells in NOD/SCID mice and CB17/SCID mice in comparison with that in BALB/c mice has not been sufficiently evaluated.

**Methods:**

Splenocytes from naïve or poly(I:C)-treated mice were isolated for phenotyping and analysis of cytotoxicity-related molecules and inhibitory receptors; for cytotoxicity assay, purified NK cells were also used.

**Results:**

The proportion of splenic NK cells did not differ significantly between NOD/SCID and CB17/SCID mice. The perforin levels in NK cells were similar between the poly(I:C)-treated CB17/SCID and NOD/SCID mice, while the granzyme B and NKG2A/C/E levels in NK cells from NOD/SCID mice were significantly lower than those from CB17/SCID mice. Moreover, the NKG2D and Ly49A levels in NK cells from NOD/SCID mice were higher than those from CB17/SCID. The splenocytes from CB17/SCID mice showed higher cytotoxicity than those from NOD/SCID mice, while the cytotoxicity of purified NK cells basically did not differ between the two strains. After *in vitro* stimulation with cytokines, the splenocytes from CB17/SCID mice showed higher IFN-*γ* production than those from NOD/SCID mice; however, NK cells did not.

**Conclusion:**

There was no significant difference in the proportion of splenic NK cells between CB17/SCID and NOD/SCID mice, and the function of NK cells was only partially compromised in NOD/SCID mice. Caution should be taken when considering the use of NOD/SCID mice as an NK-deficient model.

## 1. Introduction

As a unique member of the innate immune cells, NK cells play important roles in various immune responses, including tumor immunity [1–3], transplant rejection [4], and autoimmune diseases [5–7]. Research to obtain a better understanding of the characteristics of NK cells is underway. Some NK cells have been shown to exert adaptive immune response and are named memory or adaptive NK cells [8, 9]. In the field of biomedical research, NK cell-deficient mice are widely used and effective tools [10–14]. CB17/SCID mice, since first being reported by Bosma et al. [15] in 1983, are known to lack adaptive immune function but express normal NK cells [16–18]. NOD/LtSz-scid/scid mice (NOD/SCID) were generated as a new murine model in 1995 by Shultz et al. [19] and described as having multiple defects in innate immunity, as well as lacking both T and B cells. Since then, these mice have been used as an NK-deficient model in many studies [20–25]. Based on published data [26–28] and our unpublished experimental observations, it appears that NK cells in NOD/SCID mice may not be as deficient as previously thought. After reviewing an original paper [19], we realized that the proportion of NK cells from the NOD/SCID splenocytes was detected using NK1.1 antibody, and NK cell activity was assessed using total splenocytes as effector cells. NK1.1 is an antigen that is limitedly expressed only in some inbred mouse strains (e.g., C57BL/6 or C57BL/10); most other inbred mouse strains (e.g., BALB/c) express NK1.1 at a low level or not at all [29]. Although another research group investigated the NK cells in the placenta of pregnant NOD/SCID mice using DX5 as an NK cell marker [30], a clear technical defect undermined the credibility of the obtained data. Moreover, most of the data reported to date were obtained using splenocytes as effector cells to evaluate the cytotoxicity of NK cells in NOD/SCID mice. To clarify this discrepancy and reveal the actual status of NK cells in NOD/SCID mice, in this study, we reevaluated the NK cell activity of NOD/SCID in comparison with that of CB17/SCID and BALB/c mice.

## 2. Materials and Methods

### 2.1. Reagents

The mAbs APC anti-mouse CD49b (clone DX5) and Annexin V/7-AAD kit were obtained from BD PharMingen (San Diego, CA, USA). FITC anti-mouse CD3*ε* (clone 145-2C11), PE/CY7 anti-mouse CD19 (clone 1D3), and PE anti-mouse NK1.1 (clone PK136) were obtained from Sungene Biotech (Tianjin, China). PE/Cy7 anti-mouse CD69 (clone H1.2F3), PE/Cy7 anti-mouse NKp46 (clone 29A1.4), and FITC anti-mouse NKG2A/C/E (clone 20d5) were from eBioscience (San Diego, CA, USA). FITC anti-mouse granzyme B (clone GB11), PE anti-mouse NKG2D (clone CX5), PE anti-mouse perforin (clone S16009A), PE anti-mouse IFN-*γ* (clone XMG1.2), PE anti-mouse FasL (clone MFL3), PerCP/Cy5.5 anti-mouse TRAIL (clone N2B2), FITC anti-mouse Ly49A (clone YE1/48.10.6), and buffers for staining, fixation, and permeabilization, as well as CFSE Cell Division Tracker Kit, were from BioLegend (San Diego, CA, USA). Mouse IFN-*γ* ELISA kit was from Dakewe Biotech Co., Ltd. (Shenzhen, China). Recombinant murine IL-2, IL-12, and IL-15 were from PeproTech Inc. (Rockhill, NJ, USA). Poly(I:C) was from InvivoGen (San Diego, CA, USA). EasySep™ mouse NK cell isolation kit was from STEMCELL Technologies, Inc. (Cambridge, UK).

### 2.2. Mice

Six- to eight-week-old NOD/SCID, CB17/SCID, BALB/c, and C57BL/6 mice were obtained from Beijing Vital River Laboratory Animal Technology Co., Ltd. (Beijing, China) and maintained at Jinzhou Medical University in a Specific Pathogen Free-level Laboratory Animal Room. All experiments with animals were performed in accordance with the Guide for the Care and Use of Laboratory Animals as approved by China National Institutes of Health.

### 2.3. Analysis of Cell Surface Molecules by Flow Cytometry (FCM)

Splenocytes from each sample were suspended in 100 *μ*l of staining buffer. A cocktail of antibodies (combination of fluorescent-conjugated antibodies selected from those listed in Section 2.1) was added to the cell suspension, and then, the stained samples were incubated on ice for 20 min. After washing with phosphate-buffered saline (PBS), the stained cells were analyzed by FCM (BD Biosciences FACSCanto II, USA).

### 2.4. Analysis of Intracellular Molecules by FCM

Cells (in some experiments, cells were stained for surface markers first and then washed twice in PBS) were fixed and permeabilized for 20 min at room temperature. After washing twice in PBS, cells were suspended in staining buffer and stained with PE-perforin (or PE-IFN-*γ*) and FITC-granzyme B antibodies at room temperature for 20 min in the dark. After two more washes, the stained cells were analyzed by FCM.

### 2.5. In Vivo Stimulation of NK Cells with Poly(I:C)

NOD/SCID and CB17/SCID mice were injected intraperitoneally (i.p.) with poly(I:C) at a dosage of 100 *μ*g/20 g body weight [31] at 0 and 40 h. Splenocytes were isolated from mice at 64 h.

### 2.6. In Vitro Stimulation of NK Cells with Cytokines

Several cytokines related to NK cell function were selected from those reported earlier [32, 33] for NK cell activation experiments. The cells were stimulated with cytokines in accordance with published protocols [34–36] with some modifications. Briefly, splenocytes (1 × 10^6^) from naïve or poly(I:C)-treated mice were cultured with IL-2 (1000 U/ml), IL-12 (10 ng/ml), and IL-15 (20 ng/ml) for 24–48 h in an incubator at 37°C with a 5% CO_2_ humidified atmosphere. Monensin (×1, BioLegend) was added to the culture medium for the last 5 h of incubation.

### 2.7. Assessment of NK Cell Cytotoxicity

YAC-1 cells were labeled with 5 *μ*M CFSE for 20 min at 37°C and then washed twice in PBS. Mice were treated with poly(I:C) as mentioned above (Section 2.5). Splenocytes were taken from the mice at 64 h. NK cells were purified from the splenocytes with EasySep™ mouse NK cell isolation kit, in accordance with the manufacturer's instructions. Splenocytes or purified NK cells were stimulated with cytokines for 24 h as mentioned above (Section 2.6). After stimulation, the splenocytes were cocultured with the CFSE-labeled YAC-1 cells at effector-to-target ratios (E:T) of 100 : 1, 25 : 1, and 6.25 : 1, while the purified NK cells were cocultured with the CFSE-labeled YAC-1 cells at effector-to-target ratios (E:T) of 10 : 1, 5 : 1, 2.5 : 1, and 1.25 : 1. After 6 h of incubation at 37°C, cells were collected and washed twice in PBS and then stained with 7-AAD and detected by FCM.

### 2.8. Assay of Cytokine Production in Culture Supernatant by ELISA

The supernatants from the cytokine-stimulated splenocytes (Section 2.6) were collected, and IFN-*γ* was analyzed using a mouse IFN-*γ* ELISA kit, in accordance with the manufacturer's instructions. Briefly, diluted samples (100 *μ*l/well) were added to the microplate that had been precoated with an antibody specific for IFN-*γ* and incubated with a biotinylated antibody against IFN-*γ*. Each sample was assayed in duplicate. After 90 min of incubation at 37°C, the microplate was washed and incubated with streptavidin-HRP conjugate at 37°C for 30 min, followed by washing and incubation at 37°C for 10–20 min in a substrate solution to detect peroxidase activity before the addition of the stop solution. The absorbance was measured at 450 nm.

### 2.9. Statistical Analysis

All statistical analyses were performed using SPSS 16.0 statistical software package (SPSS, Inc., Chicago, IL, USA). All data were from at least three independent experiments, and the presented data are expressed as mean ± SD. Statistical analysis was carried out by one-way ANOVA. Values of *P* < 0.05 and *P* < 0.01 were considered statistically significant.

## 3. Results

### 3.1. Verification of NK1.1 Expression in Different Mouse Strains

**Figure 1:**
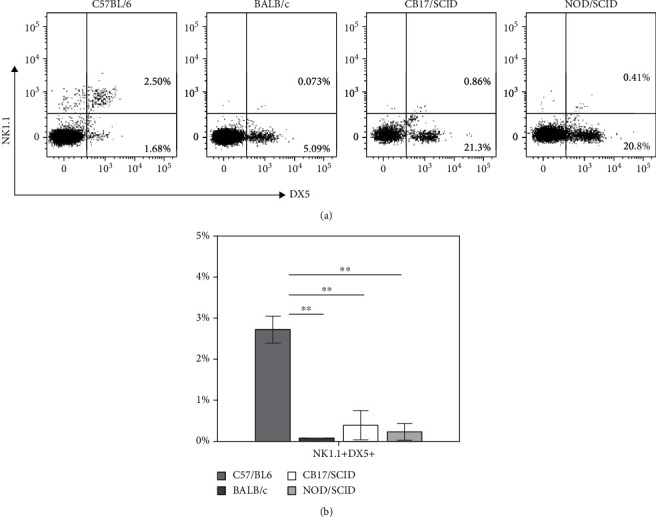
The expression of NK1.1 in different mouse strains. The splenocytes were isolated from naïve mice, stained with fluorescence-conjugated antibodies to NK1.1 and DX5, and analyzed by FCM. (a) Representative FCM histogram shows the percentages of NK1.1^+^DX5^+^ cells gated on CD3^−^ splenocytes. (b) Statistical analysis of the percentages of NK1.1^+^DX5^+^ cells. Results are presented as mean ± SD. *n* = 6 mice per group. Values of ^∗∗^*P* < 0.01 were considered statistically significant. Three independent experiments were performed, and representative data are shown.

To verify the expression of NK1.1 in NOD/SCID mice, we compared the expression of NK1.1 and DX5 on splenocytes in different mouse strains as indicated. The results showed that NK1.1^+^DX5^+^ cells were prominent in C57BL/6 mice. In contrast, almost no NK1.1^+^DX5^+^ cells were observed in BALB/c, CB17/SCID, and NOD/SCID mice (Figure 1).

### 3.2. Assessment of T, B, and NK Cells in Naïve Mice

**Figure 2:**
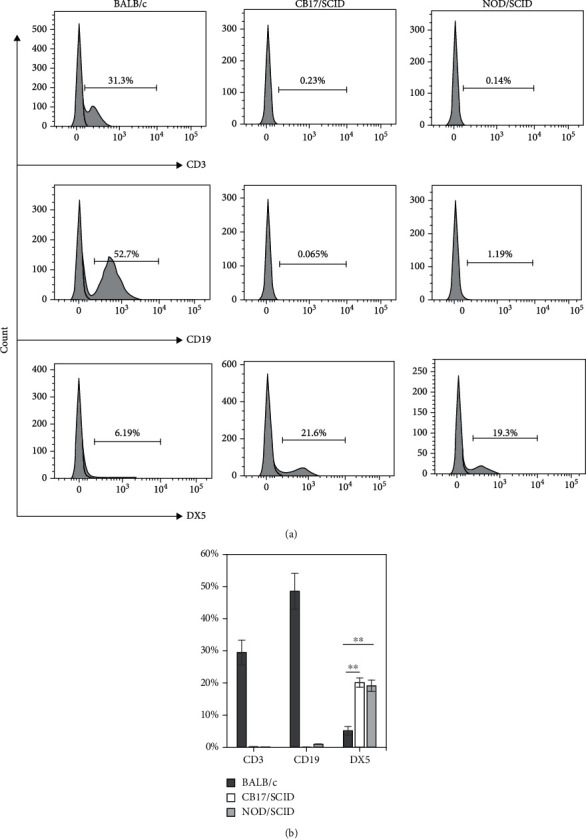
Analyses of T, B, and NK cells in splenocytes from naïve mice. The splenocytes were stained with fluorescence-conjugated antibodies to CD3, CD19, and DX5 and analyzed by FCM. (a) Representative FCM histograms show the percentages of CD3-, CD19-, and DX5-positive cells gated on all splenocytes. (b) Statistical analysis of the percentages of CD3-, CD19-, and DX5-positive cells. Results are presented as mean ± SD. *n* = 6 mice per group. Values of ^∗∗^*P* < 0.01 were considered statistically significant. Three independent experiments were performed, and representative data are shown.

T- and B-cell deficiencies were confirmed by comparing the percentages of CD3^+^ and CD19^+^ cells in the splenocytes from BALB/c with those from CB17/SCID and NOD/SCID mice. The percentages of NK cells in CB17/SCID and NOD/SCID mice were higher than those in BALB/c mice, as determined by comparing DX5^+^ cells, but there were no significant differences (*P* > 0.05) in these percentages between CB17/SCID and NOD/SCID mice (Figure 2).

### 3.3. Analyses of Cytotoxicity-Related Molecules and Inhibitory Receptors in/on Splenic NK Cells from Naïve Mice

**Figure 3:**
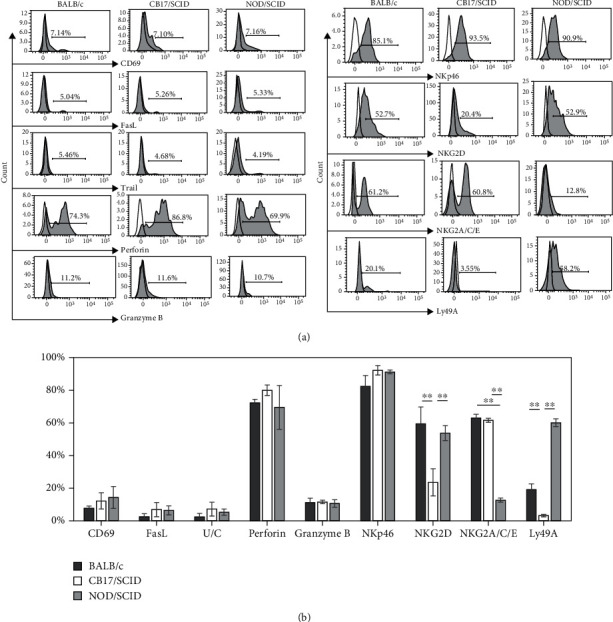
The expression of cytotoxicity-related molecules and inhibitory receptors in/on splenic NK cells from naïve mice. (a) Representative FCM data showing the CD69, FasL, TRAIL, perforin, granzyme B, NKp46, NKG2D, NKG2A/C/E, and Ly49A expression gated on splenic DX5^+^ NK cells. (b) Histogram shows the statistical analysis of FCM data for CD69, FasL, TRAIL, perforin, granzyme B, NKp46, NKG2D, NKG2A/C/E, and Ly49A expression. Results are presented as mean ± SD. *n* = 6–8 mice per group. Values of ^∗∗^*P* < 0.01 were considered statistically significant. Three independent experiments were performed, and representative data are shown.

Splenocytes from naïve BALB/c, CB17/SCID, and NOD/SCID mice were stained for surface or intracellular molecules and analyzed with FCM gating on DX5^+^ splenic NK cells. The expression of CD69, FasL, TRAIL, and NKp46 and the production of granzyme B and perforin did not show significant differences among the three mouse strains, but the expression of NKG2D on the splenic NK cells from CB17/SCID was significantly lower than that from BALB/c and NOD/SCID mice. The expression of inhibitory receptor NKG2A/C/E on the splenic NK cells from CB17/SCID and BALB/c was significantly higher than that from NOD/SCID mice, while the expression of Ly49A on the splenic NK cells of NOD/SCID mice was significantly higher than that of CB17/SCID and BALB/c mice (Figures 3(a) and 3(b)).

### 3.4. Assessment of Killing Capacity and Cytotoxicity-Related Molecules and Inhibitory Receptors Expression in/on NK Cells from Poly(I:C)-Treated Mice

**Figure 4:**
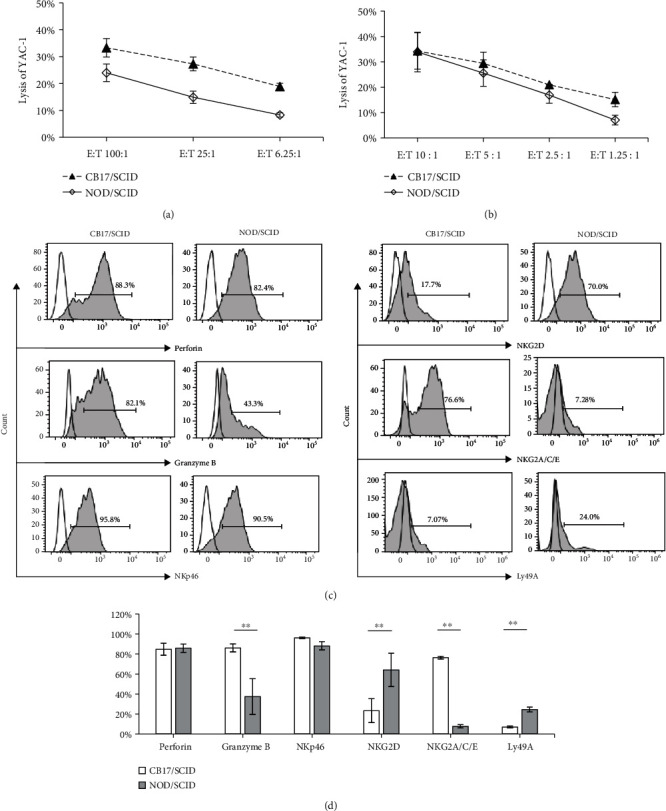
The expression of cytotoxicity-related molecules, inhibitory receptors, and killing capacity of NK cells from poly(I:C)-treated mice. Splenocytes or purified NK cells from poly(I:C)-treated mice were stimulated with cytokines and then mixed with CFSE-labeled YAC-1 cells at different E:T ratios as indicated. (a) The killing activity of the splenocytes. (b) The killing activity of the purified NK cells. (c) FCM analyses of perforin, granzyme B, NKp46, NKG2D, NKG2A/C/E, and Ly49A expression gated on splenic DX5^+^ NK cells. Histograms show representative data from three independent experiments. (d) Histogram shows the statistical analysis of FCM data for the expression of perforin, granzyme B, NKp46, NKG2D, NKG2A/C/E, and Ly49A. Results are presented as mean ± SD. *n* = 6 mice per group. Values of ^∗∗^*P* < 0.01 were considered statistically significant.

Splenocytes and purified NK cells from poly(I:C)-treated mice were stimulated *in vitro* with cytokines for 24 h and then mixed with target cells at different E:T ratios (Section 2.7). As shown in Figure 4, the cytotoxicity of splenocytes from the CB17/SCID mice was significantly higher than that from the NOD/SCID mice; that is, the percentages of the target cells lysed by the effector cells from NOD/SCID mice were about one-half to two-thirds of that from CB17/SCID mice at each E:T ratio (Figure 4(a)). However, the cytotoxicity of purified NK cells showed no significant interstrain difference in the percentages of lysed target cells at all of the E:T ratios except for the lowest one (Figure 4(b)). In the analysis of cytotoxicity-related molecules by gating on splenic DX5^+^ NK cells, no significant differences in the expression of NKp46 or the production of perforin were observed between the two mouse strains, whereas the production of granzyme B was significantly higher in the splenic NK cells from CB17/SCID than that from NOD/SCID mice. In addition, the expression of NKG2D was much higher on the splenic NK cells from NOD/SCID than that from CB17/SCID mice. The expression of inhibitory receptor NKG2A/C/E on the splenic NK cells from CB17/SCID was significantly higher than that from NOD/SCID mice, while the expression of Ly49A on the splenic NK cells from NOD/SCID was significantly higher than that from CB17/SCID mice (Figures 4(c) and 4(d)).

### 3.5. Analysis of IFN-*γ* In Vitro

**Figure 5:**
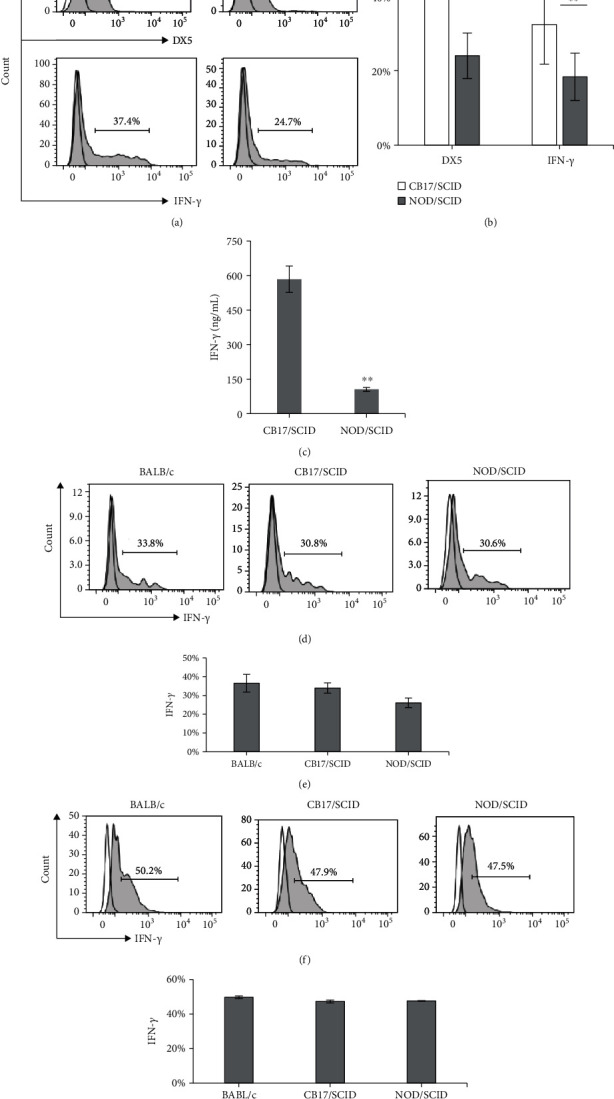
Assay of IFN-*γ* in cells and cell culture supernatant *in vitro*. The splenocytes from naïve (a–e) or poly(I:C)-treated (f, g) mice were cultured with cytokines for 48 h. (a) Representative FCM data show IFN-*γ* in and the expression of DX5 on the splenocytes from CB17/SCID and NOD/SCID mice. (b) Statistical analysis of the FCM data for IFN-*γ* and DX5 expression. (c) Statistical analysis of the ELISA data for IFN-*γ* in the culture supernatant. (d) Representative FCM data show the IFN-*γ* expression gated on DX5^+^ populations of the splenocytes from naïve mice. (e) Statistical analysis of the FCM data for the IFN-*γ* expression on splenic DX5^+^ populations. (f) Representative FCM data show the IFN-*γ* expression gated on DX5^+^ populations of the splenocytes from poly(I:C)-treated mice. (g) Statistical analysis of the FCM data for the IFN-*γ* expression on DX5^+^ populations of the splenocytes from poly(I:C)-treated mice. FCM histograms show representative data of three independent experiments. Results are presented as mean ± SD. *n* = 6 mice per group. Values of ^∗∗^*P* < 0.01 were considered statistically significant.

Splenocytes from naïve or poly(I:C)-treated mice were stimulated with cytokines (IL-2, IL-12, IL-15) for 48 h before the analysis of IFN-*γ* in cells by FCM and in supernatant by ELISA. As shown in Figures 5(a) and 5(b), the production of IFN-*γ* and the percentage of DX5^+^ cells among the cytokine-stimulated splenocytes from naïve CB17/SCID mice were higher than those from naïve NOD/SCID mice. The analysis of IFN-*γ* in the culture supernatant was in line with that in the splenocytes (Figure 5(c)); that is, the level from CB17/SCID was significantly higher than that from NOD/SCID mice. Meanwhile, the expression level of IFN-*γ* in splenic NK cells (gated on the DX5^+^ population of splenocytes) showed no statistically significant interstrain difference.

## 4. Discussion

In this study, we investigated the proportion and activity of splenic NK cells in BALB/c, CB17/SCID, and NOD/SCID mice in order to reevaluate NOD/SCID mice as an NK cell-deficient model. CB17/SCID mice are a BALB/c stock congenic for a C57BL/Ka Igh allotype and deficient in adaptive immunity [19]. NOD/SCID mice were developed in order to transfer the scid mutation from CB-17 congenic mice to diabetes-susceptible nonobese diabetic (NOD) mice [37]. Therefore, at least in part, both CB17/SCID and NOD/SCID mice have a BALB/c background, and NOD/SCID mice acquire immunological defects from both their CB17/SCID and NOD backgrounds. Although the lack of expression of NK1.1 on the NK cells in BALB/c mice is well known [29, 38, 39], considering that NOD/SCID mice were generated by Shultz et al. [19] and they evaluated NK cells in NOD/SCID mice by targeting NK1.1, we confirmed the status of NK cells by targeting DX5 and NK1.1 in BALB/c, C57BL/6, CB17/SCID, and NOD/SCID mice. As we expected, a clear NK1.1^+^ cell population was detected only in C57BL/6 mice, while the DX5^+^ populations were detected in all of the four mouse strains (Figure 1), indicating that the data from the flow cytometry analyses of NOD/SCID splenic NK cells reported by Shultz et al. [19] are inadequate.

To further ascertain the proportion of NK cells in NOD/SCID mice and ensure that the mice had no “leaky” SCID [40] in this assay, we assessed the splenic NK cells by FCM using DX5 as an NK cell marker and confirmed the existence of adaptive immune cells using BALB/c mice as a control. Although lower percentages of DX5^+^ cells were observed in the splenocytes from some NOD/SCID mice, the statistical analysis showed no significant difference in the proportion of splenic DX5^+^ cells between the NOD/SCID and CB17/SCID mice. The significantly low CD3 and CD19 signals in the splenocytes from NOD/SCID and CB17/SCID mice confirmed the reliability of the results (Figure 2). Notably, a report has shown that 32% of the splenic population from CB17/SCID mice were NK1.1-positive for the NK cells compared with 20% of that from NOD/SCID mice [41]. We do not know the reason for this discrepancy, except that the mice that they used were produced from their own breeding colony, and the mouse anti-mouse NK1.1 antibody was phycoerythrin-conjugated and obtained from Serotec Ltd. (Kidlington, UK).

To evaluate the functional activity of NK cells in NOD/SCID mice, we detected the expression of cytotoxicity-related molecules on/in NK cells by FCM (Figure 3). Our data showed that the expression levels of CD69, FasL, TRAIL, granzyme B, and perforin on/in the splenic NK cells from naïve mice were all similar among the three mouse strains, except that the NK cells from NOD/SCID mice and BALB/c mice even expressed a higher level of NKG2D than those from CB17/SCID mice. This suggested that the cytotoxicity of NK cells in NOD/SCID mice could be no less than that in CB17/SCID mice. Based on these findings and considering that the NK cell cytotoxicity was assessed by Shultz et al. using splenocytes from poly(I:C)-treated mice, we adopted their protocol and compared the cytotoxicity with that of purified NK cells. The cytotoxicity of splenocytes from the CB17/SCID mice was significantly higher than that from the NOD/SCID mice (Figure 4(a)). This is in line with a report by Shultz et al. [19]. However, the cytotoxicity of purified NK cells showed no significant interstrain difference in the percentages of lysed target cells at all of the E:T ratios except for the lowest one (Figure 4(b)). To obtain a better understanding of this discrepancy, we checked the expression of cytotoxicity-related molecules on/in the cytokine-stimulated NK cells from the poly(I:C)-treated mice (Figure 4(c)). Statistical analysis of the FCM data showed no significant difference in the production of perforin among the two mouse strains, while significantly higher levels of granzyme B were observed in NK cells from CB17/SCID mice than that from NOD/SCID mice. Meanwhile, the expression of NKG2D on NK cells was significantly higher in NOD/SCID than that in CB17/SCID mice (Figure 4(d)). As poly(I:C) can induce macrophages and dendritic cells to produce cytokines that subsequently activate NK cells [42, 43], and the macrophages and dendritic cells in NOD/SCID mice are functionally less mature than those in CB17/SCID [19], we postulate that the discrepancy between the cytotoxicities of total splenocytes and the purified NK cells could be explained by the contribution of cytotoxic factors other than NK cells in splenocytes. As for the similarity in the cytotoxicity of purified NK cells from the two strains, multiple factors might be involved. The major mechanism of granzyme B in NK cell killing is through cooperation with perforin. Therefore, a low level of perforin could limit the function of high-level granzyme B. According to published data, NKG2A and Ly49s may act synergistically in regulating NK cell function. Members of the Ly49 family are critical for NK cell education, while NKG2A is required for such education particularly when the Ly49 family is absent. Ly49 signaling may influence the NK cell receptor repertoire, while NKG2A deficiency mildly impairs NK cell activity [44]. Therefore, although there is a significantly higher level of granzyme B and a lower level of Ly49A expression in NK cells from CB17/SCID mice, it is possible that the significantly higher level of NKG2D and the lower level of NKG2A/C/E in NOD/SCID mice as well as the similar expression of killer receptor NKp46 and the production of perforin may work together to minimize or balance the differences of the above functional factors between the two mouse strains. However, considering the complexity of the functional molecules in NK cell regulation, we cannot rule out the possibility that other factors may be involved in the cytotoxicity that we observed. Thus, we cannot draw definitive conclusions on this issue based on our current experimental data.

We identified the discrepancy in the killing activity of the purified NK cells at higher vs. lower E:T ratios and speculate that the killing ability may be more dependent on granzyme B when there are fewer effector cells. Of course, more factors may contribute to the activation and function of NK cells, and an interesting remaining question is how those functional molecules are integrated during NK cell killing, which needs further investigation.

To obtain a better understanding of the function of NK cells in the two mouse strains, we evaluated the major cytokine production of the NK cells. Early studies showed that poly(I:C) was able to stimulate IL-12 production with subsequent NK cell activation and improved IFN-*γ* production [45], and that cytokines secreted by NKL-IL15 cells, including IFN-*γ*, could induce the expression of NKG2D ligands on target cells and thus increase the susceptibility of leukemia cells to NK cell-mediated cytolysis [23]. Therefore, we assessed the IFN-*γ* production of total splenocytes and splenic NK cells from naïve and poly(I:C)-treated mice for strain comparison. As shown in Figure 5, when the total splenocytes from naïve mice were stimulated with cytokines (IL-2, IL-12, IL-15), both the proportion of NK cells and IFN-*γ* production were significantly higher in the splenocytes from CB17/SCID than those from NOD/SCID mice; however, when we focused on the NK cells by gating on the DX5^+^ population, IFN-*γ* production showed no significant difference among these three mouse strains. These findings further suggest that the function of NK cells in NOD/SCID mice is a complex trait under multigenic control and, although it is not fully comparable with that in BALB/c, it is only partially compromised.

We noticed the reported data that the proportion of DX5^+^ NK cells in the placentas from NOD/SCID mice was significantly lower than that from BALB/c mice, and the NK cells strikingly increased after the poly(I:C) treatment of NOD/SCID mice [30]. Supposing that their FCM data are still valid after a compensation correction, based on our data, a possible explanation for this could be that there were more functional molecules in BALB/c mice that could assist in the activation and migration of NK cells and thus be detected in some specific location at a high level. However, investigation of the distribution of NK cells in organs other than the spleen was outside the remit of this work, so it is not possible to address this matter in the current study. Another notable point is that, as indicated in a recent report by Flavell et al. [24], in previous studies, anti-asialo GM1 antibody was used to deplete NK cells in vivo to comparatively evaluate the cytotoxicity of NK cells in NOD/SCID vs. CB17/SCID or other mouse strains. However, the authors asserted that NK cells are likely to be the major, but not necessarily the sole, participating cytotoxic effector cell. Our findings in the current study, together with the functionally compromised macrophages, complement, and some other adaptive immunocomponents in NOD/SCID mice, further invalidate those evaluation models.

## 5. Conclusion

There is no significant difference in the proportion of splenic NK cells between CB17/SCID and NOD/SCID mice, and the function of NK cells is only partially compromised in NOD/SCID mice. Thus, one should be very cautious when using NOD/SCID mice as an NK cell-deficient model.

## Data Availability

The data generated or analyzed during this study are included in this article. Raw data supporting the findings of this study are available from the corresponding author upon reasonable request.
